# A Rare Cause of Liver Cirrhosis and Gastritis: Primary Gastrointestinal Amyloidosis in a Patient With Multiple Myeloma

**DOI:** 10.7759/cureus.24858

**Published:** 2022-05-09

**Authors:** Davood K Hosseini, Kristen Delcalzo, Antik Patel, Raul Rodriguez, Nilesh B Shukla

**Affiliations:** 1 Department of Internal Medicine, Hackensack University Medical Center, Hackensack, USA; 2 School of Medicine, St. George's University, True Blue, GRD; 3 Pathology and Laboratory Medicine, Rutgers Robert Wood Johnson Medical School, New Brunswick, USA; 4 Department of Gastroenterology and Hepatology, Hackensack University Medical Center, Hackensack, USA

**Keywords:** abdominal pain with nausea and vomiting, monoclonal antibody, multiple myeloma, liver cirrhosis, amyloidosis

## Abstract

A 65-year-old male with a 25-year history of chronic alcoholism presented to the emergency department for a two-week history of non-radiating right upper quadrant abdominal pain associated with pruritus, nausea, coffee-ground emesis, and clay-colored stools. The exam was notable for icteric sclera, right upper quadrant abdominal tenderness, ascites, and hepatomegaly. Initial workup revealed new-onset unexplained elevated liver enzyme. The CT scan revealed diffuse liver cirrhosis, periportal lymphadenopathy, and stigmata of portal hypertension including hepatosplenomegaly, ascites, and varices. Esophagogastroduodenoscopy (EGD) with endoscopic ultrasound was performed, which showed gastritis and enlarged porta hepatis, which was ultimately biopsied and revealed extracellular amyloid deposition in peri-sinusoidal spaces consistent with amyloidosis. Transesophageal echocardiogram raised suspicion for cardiac involvement with amyloid deposit.

The patient was started on steroids and chemotherapy with daratumumab, however his condition was complicated by septic shock, which led to an admission in the ICU followed by endotracheal intubation and multi-organ failure and eventual palliative care. Our case highlights the importance of clinical suspicion of GI amyloidosis in patients with constitutional symptoms including fatigue, weight loss, and unexplained liver disease. Once amyloidosis is considered, the diagnosis can be obtained by tissue biopsy from either the GI tract or subcutaneous adipose tissue.

## Introduction

Amyloidosis is a heterogeneous group of diseases, characterized by extracellular deposition of amyloids, insoluble aggregates of misfolded proteins with twisted β-pleated sheet tertiary structure, resulting in disruption of tissue structure and function. The most common form, systemic amyloidosis, is characterized by production of amyloidogenic precursor proteins at a site remote from the organs of amyloid deposition [1].

The abstract has been accepted by the official Journal of the American College of Gastroenterology (ACG).

## Case presentation

A 65-year-old male with a 25-year history of chronic alcoholism presented to the emergency department for a two-week history of non-radiating right upper quadrant abdominal pain associated with pruritus, nausea, coffee-ground emesis, and clay-colored stools. He also reported weakness, fatigue and a 10-pound unintentional weight loss over four months. The exam was notable for icteric sclera, right upper quadrant abdominal tenderness, ascites, and hepatomegaly. Initial workup revealed new-onset unexplained elevated liver enzyme (aspartate transaminase [AST] 85 U/L, alanine transaminase [ALT] 32 U/L, total bilirubin 3.4 mg/dL, alkaline phosphatase [ALP] 563 U/L).

**Figure 1:**
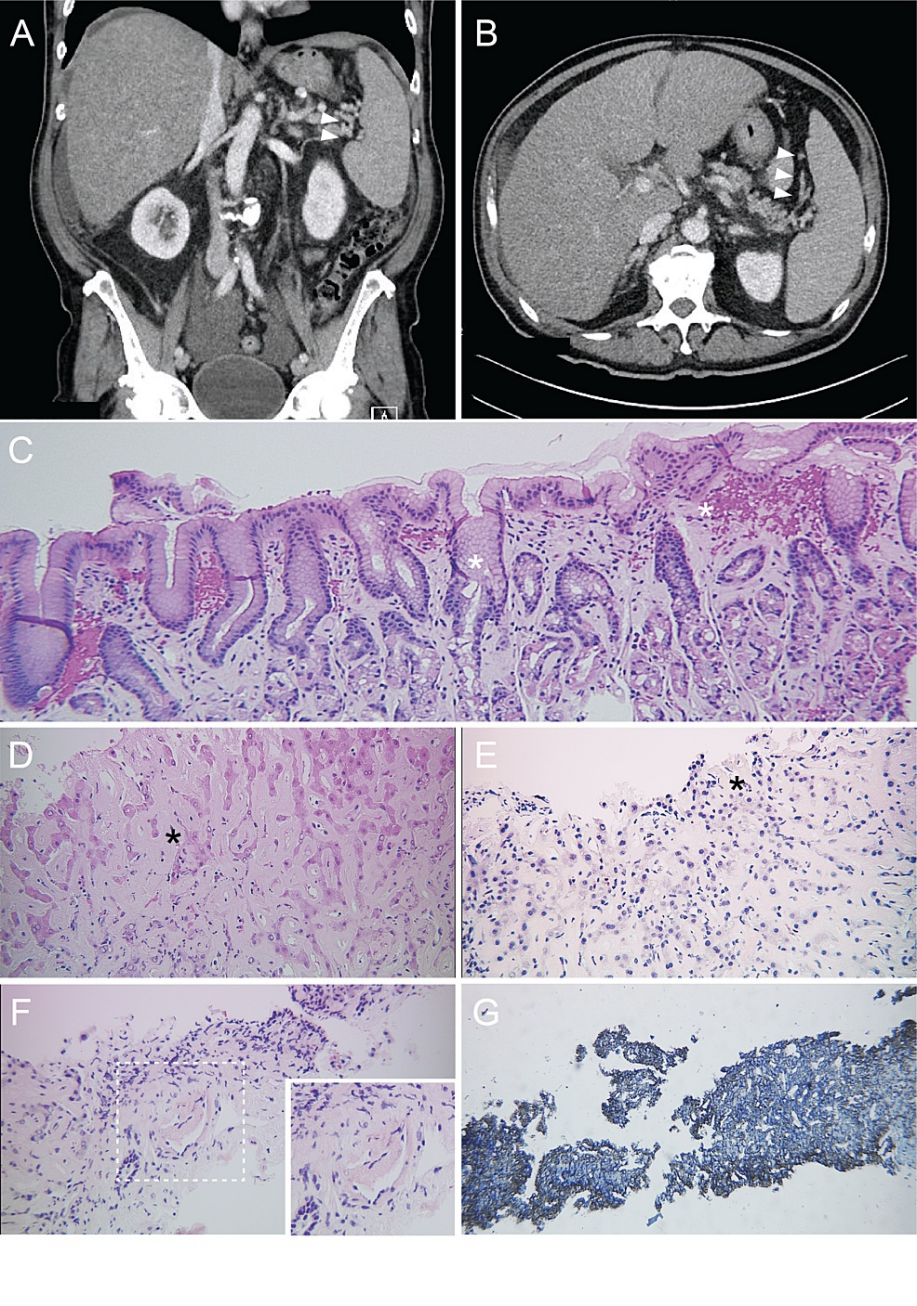
CT scan CT scan (A-B) showing hepatosplenomegaly and peri-splenic varices (arrowheads). Pathologic findings (C-G) of AL-type amyloidosis of the luminal gastrointestinal tract. C. Histologic examination (Hematoxylin-Eosin staining) of a gastric antral type mucosa with chronic inactive gastritis and interstitial deposition of pink amorphous material (white asterisk). Histologic examination of liver with amorphous material deposition: Hematoxylin-Eosin staining (black asterisk in D-E) and crystal violet staining (G). F. Vascular wall are almost always involved first in the systemic amyloidosis (Hematoxylin-Eosin staining).

Abdominal ultrasound was significant for hepatomegaly with perihepatic ascites. The computerized tomography (CT) scan revealed diffuse liver cirrhosis, periportal lymphadenopathy, and stigmata of portal hypertension including hepatosplenomegaly, intra-abdominal ascites, and varices (Figure 1).

Esophagogastroduodenoscopy with endoscopic ultrasound (EGD with EUS) was performed, which showed gastritis and enlarged porta hepatis, which was ultimately biopsied and sent for cytology and pathology (Figure 1C). Given the unexplained elevation of the liver enzymes, patient underwent liver biopsy which revealed extracellular amyloid deposition in peri-sinusoidal spaces consistent with amyloidosis.

**Figure 2:**
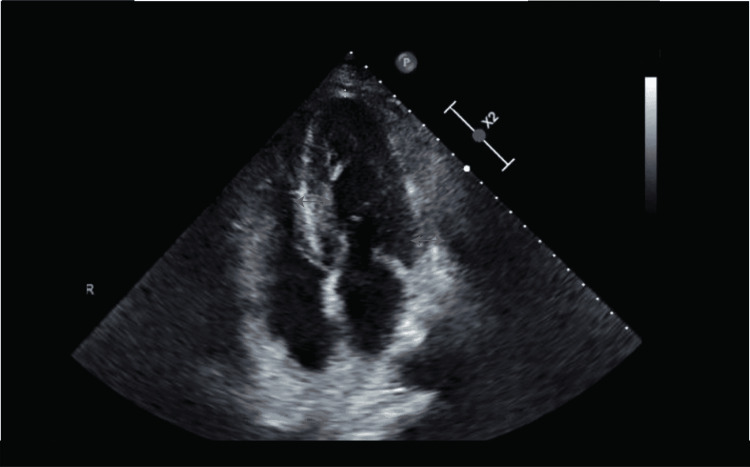
Transthoracic echocardiogram Apical 4 chamber view demonstrating increased septal wall thickness (red arrow), and lateral left ventricle wall (green arrow), with apical sparing (yellow arrow).

This finding prompted further workup with a transesophageal echocardiogram which raised suspicion for cardiac involvement with amyloid deposit (Figure 2). The patient declined an endo-myocardial biopsy, precluding a definitive diagnosis. 

Immunoglobulin levels were subsequently investigated and showed elevated IgG antibodies. A K/L free ratio was elevated to 94.7. A myeloma flow cytometry exhibited an IgG kappa monoclonal-gammopathy. Finally, a CT-guided bone marrow biopsy from the iliac crest was obtained, confirming an IgG kappa multiple myeloma with an 80% proportion of plasma cells (DS 2A, ISS 3). 

The patient was started on steroids and chemotherapy with daratumumab, however his condition was complicated by septic shock, which led to admission to the ICU followed by endotracheal intubation, multi-organ failure and eventual palliative care.

## Discussion

Acquired systemic amyloidosis is more frequently seen in patients with underlying clonal plasma cell dyscrasias, whereas immunoglobulin light chain (AL) amyloidosis typically involves the kidney (74%) and heart (60%) [2]. While proteinuria and nephrotic syndrome are the main presentations of renal amyloidosis, systolic or diastolic dysfunction and arrhythmia are the main presenting features of cardiac amyloidosis.

In contrast, primary amyloidosis of the gastrointestinal (GI) tract is a rare entity defined by the presence of GI symptoms requiring direct biopsy for confirmation. Clinically dominant hepatic amyloidosis is characterized by unusual accumulation of amyloids in the liver producing hepatomegaly in 57-83% of patients as well as moderate jaundice and cholestasis [3]. The main treatment modality for amyloidosis is to suppress the abundance of the amyloidogenic precursor protein formation, combination of chemotherapy, and hematopoietic stem cell transplantation [2].

Localized GI and hepatic amyloidosis have rarely been described in the literature. This condition poses great diagnostic challenges and warrants extensive workup before definitive diagnosis [4,5]. AL amyloidosis has a grim prognosis given the association with an underlying malignancy. Among them, patients with GI involvement have a worse prognosis compared to those without GI involvement (survival time of 7.9 months and 15.8 months, respectively) [6].

## Conclusions

Our case highlights the importance of clinical suspicion of GI amyloidosis in patients with constitutional symptoms including fatigue, weight loss, and unexplained liver disease. Once amyloidosis is considered, the diagnosis can be obtained by tissue biopsy from either the GI tract or subcutaneous adipose tissue. Prognosis of amyloidosis depends on the underlying etiology.
